# Metal- and UV- Catalyzed Oxidation Results in Trapped Amyloid-β Intermediates Revealing that Self-Assembly Is Required for Aβ-Induced Cytotoxicity

**DOI:** 10.1016/j.isci.2020.101537

**Published:** 2020-09-06

**Authors:** Mahmoud B. Maina, Gunasekhar Burra, Youssra K. Al-Hilaly, Kurtis Mengham, Kate Fennell, Louise C. Serpell

**Affiliations:** 1 Sussex Neuroscience, School of Life Sciences, University of Sussex, Falmer, Brighton, East Sussex BN1 9QG, UK; 2 College of Medical Sciences, Yobe State University, Yobe, Nigeria; 3 Chemistry Department, College of Science, Mustansiriyah University, Baghdad, Iraq

**Keywords:** Biochemical Mechanism, Molecular Neuroscience, Neurotoxicology

## Abstract

Dityrosine (DiY), via the cross-linking of tyrosine residues, is a marker of protein oxidation, which increases with aging. Amyloid-β (Aβ) forms DiY *in vitro* and DiY-cross-linked Aβ is found in the brains of patients with Alzheimer disease. Metal- or UV- catalyzed oxidation of Aβ42 results in an increase in DiY cross-links. Using DiY as a marker of oxidation, we compare the self-assembly propensity and DiY cross-link formation for a non-assembly competent variant of Aβ42 (vAβ) with wild-type Aβ42. Oxidation results in the formation of trapped wild-type Aβ assemblies with increased DiY cross-links that are unable to elongate further. Assembly-incompetent vAβ and trapped Aβ assemblies are non-toxic to neuroblastoma cells at all stages of self-assembly, in contrast to oligomeric, non-cross-linked Aβ. These findings point to a mechanism of toxicity that necessitates dynamic self-assembly whereby trapped Aβ assemblies and assembly-incompetent variant Aβ are unable to result in cell death.

## Highlights

• Metal- (Cu^2+^ H_2_0_2_) or UV- catalyzedoxidation results in dityrosine (DiY) formation
• Oxidation results in DiY cross-link formation in Aβ and halts further assembly
• Non-assembling Aβ (trapped Aβ or variant Αβ monomer) is not cytotoxic

## Graphical Abstract

**Figure:**
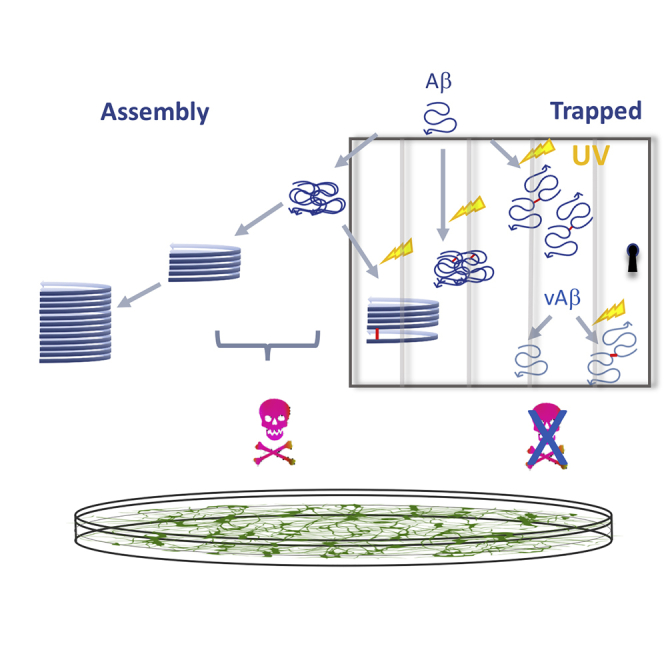

## Introduction

Alzheimer disease (AD) is the most common form of dementia, and it is characterized by the deposition of amyloid-β (Aβ) and Tau in extracellular plaques and intracellular neurofibrillary tangles, respectively. The amyloid cascade hypothesis implicates the pathological accumulation of Aβ and its aggregation from monomers into oligomers and fibrils, as a key event in the development of AD (Hardy and Higgins, 1992). Many pieces of evidence have subsequently shown that the oligomeric form of Aβ is the most toxic species, resulting in a reformulated amyloid cascade hypothesis in which Aβ oligomers are proposed to be central to AD pathogenesis (Selkoe and Hardy, 2016). Indeed, accumulated evidence shows that Aβ oligomers disrupt cellular function in cultured cells and animal models (Marshall et al., 2016; Lambert et al., 1998; Lacor et al., 2007; Zhang et al., 2014; Selkoe and Hardy, 2016). Numerous studies have searched for the elusive “toxic” species and tried to characterize its structure. For example, 12mers, ∗56 KDa, and hexamers have all been implicated as specific structural species that interact with particular receptors (e.g., NDMA) leading to downstream cell death (Lesne et al., 2006; Reed et al., 2011).

Oxidative stress has been proposed as a key mechanism that mediates Aβ toxicity (Butterfield and Halliwell, 2019; Butterfield et al., 2013) and, it is potentially one of the earliest sources of damage in human AD (Nunomura et al., 2001). Furthermore, using a cellular model, we have shown that oxidative stress is one of the earliest events induced by Aβ oligomers (Maina et al., 2018). One of the ways that oxidative stress causes cellular damage is through protein oxidation. The most common consequences of protein oxidation include amino acid side-chain modification, protein fragmentation, and protein cross-linking (e.g., via dityrosine [DiY] bond formation) (Lund et al., 2011). DiY cross-linking is mediated via carbon-carbon bonding between two proximal tyrosines, resulting in a stable, non-reversible covalent bond (Gross and Sizer, 1959). DiY cross-linking is known to provide elasticity, strength, and stability to proteins and has been shown to form within proteins involved with neurodegenerative diseases (e.g., Aβ and α-synuclein) (Galeazzi et al., 1999; Souza et al., 2000). Indeed, we have previously shown the colocalization of DiY with Aβ in plaques and α-synuclein in Lewy bodies in human AD and Parkinson disease brain tissue, respectively (Al-Hilaly et al., 2013, 2016). In addition to DiY, other modifications such as oxidation of histidine, lysine, and methionine35 (met35) have been shown to occur in Aβ (Kowalik-Jankowska et al., 2004; Ali et al., 2005; Palmblad et al., 2002; Friedemann et al., 2015), whereas hydroxylated phenylalanine has been suggested to form cross-links alongside DiY (Zhang et al., 2019). This indicates the potential relevance of these modifications in disease pathogenesis or as markers of disease progression in AD.

To learn more about the importance of DiY cross-linking in AD specifically, several studies have investigated the impact of DiY bond formation on Aβ aggregation and toxicity, mostly using metal-catalyzed oxidation (e.g., Cu^2+^/H_2_O_2_) (MCO) and peroxidase-catalyzed oxidation. Some studies have shown that DiY cross-linking of both Aβ40 and Aβ42 results in the generation of toxic Aβ assemblies with reduced assembly speed (Barnham et al., 2004; Smith et al., 2006; Kok et al., 2013; O'malley et al., 2014, 2016; Sitkiewicz et al., 2014). Others have implicated DiY in the inhibition of Aβ40 assembly, especially in highly oxidative environments (Gu et al., 2018) and it has been demonstrated to be associated with the formation of non-amyloidogenic Aβ42 aggregates when catalyzed with a high concentration of Cu^2+^ (Smith et al., 2007). However, whether DiY cross-linking is a driver, facilitator, or inhibitor of Aβ self-assembly remains unclear. Moreover, given that Aβ self-assembly is rapid, the time point of the cross-linking during the assembly process may influence the nature of the cross-linked Aβ assemblies. Here, using MCO and UV-induced oxidation to induce DiY cross-linking, we show that the oxidation process results in the stabilization of Aβ42 assemblies and either prevents or very significantly slows further elongation. To specifically compare the influence of DiY cross-linking on Aβ assembly, we compared the effect of oxidation on a non-assembly competent variant Aβ (vAβ) (Marshall et al., 2016) and revealed that oxidation and DiY cross-linking does not induce or promote its assembly. We show that in the absence of H_2_O_2_, CuCl_2_ at a concentration similar to the concentration found around Aβ plaques (~400 μM) (Lovell et al., 1998) is sufficient to facilitate the DiY cross-linking and formation of Aβ42 oligomers into a long-lived oligomer population. A cell live/dead assay revealed that, unlike the self-assembling non-cross-linked Aβ, oxidized DiY containing Aβ42 is non-toxic to neuron-like cells. Our results suggest that under certain conditions *in vitro*, oxidation can result in trapping of intermediate species, which cannot elongate further and that are non-toxic to neuroblastoma cells. Together with the observation that non-assembly-competent variant Aβ is non-toxic, this reinforces the importance of a continual self-assembly process in mechanisms of Aβ toxicity.

## Results

### *In Vitro* Metal-Catalyzed Oxidation Results in the Formation of Dityrosine in Wild-Type and vAβ Peptides

To investigate the influence of oxidation on Aβ assembly, we compared the effect of MCO using CuCl_2_ and H_2_O_2_ on wild-type Aβ42 and variant Aβ42 (henceforth called Aβ and vAβ, respectively) (see Transparent Methods in Information for Authors for more details).

**Figure 1:**
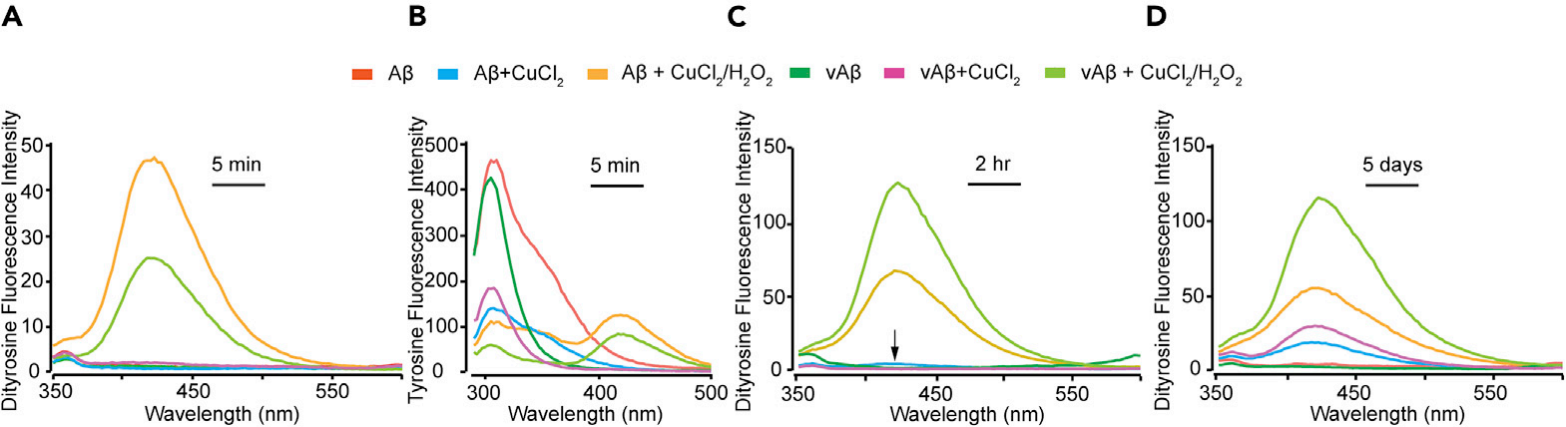
DiY Formation in Aβ and vAβ via Metal-Catalyzed Oxidation (A–D) Freshly prepared Aβ and vAβ (50 μM) were incubated at 37°C, without CuCl_2_, with CuCl_2_ (400 μM), or CuCl_2_ in combination with H_2_O_2_. Fluorescence spectra were collected 5 min post-incubation using fluorescent excitation wavelength of 320 nm and emission collected between 340 and 600 nm, with DiY peak signal observed between 400 and 420 nm (A). Fluorescence spectra were also collected at 5 min using an excitation wavelength of 280 nm and emission collected between 290 and 500, with peak tyrosine signal observed at 305 nm (B). Fluorescence spectra were collected again at 2 h (C) and then 5 days (D) to follow DiY formation over time. A minimum of three independent experiments was repeated to ensure the reproducibility of the findings.

DiY serves as a useful measure of the levels of oxidation and was used here to follow oxidation effects. Other side chains can be oxidized such as Met35, which can be detected using matrix-assisted laser desorption ionization mass spectrometry (Friedemann et al., 2015). We have previously detected DiY cross-links in Aβ and α-synuclein using a combination of techniques and shown that DiY can reliably be detected using fluorescence spectroscopy (Al-Hilaly et al., 2013, 2016). Here, rapid formation of DiY was detected for Aβ and vAβ samples that were incubated with both CuCl_2_ and H_2_O_2_ (Aβ/CuCl_2_/H_2_O_2_ and vAβ/CuCl_2_/H_2_O_2,_ henceforth called MCO), indicated by the observation of a fluorescence peak at 410 nm after only 5 min. In contrast, samples incubated with CuCl_2_ alone or with buffer-only showed no peak at 410 nm (Figure 1A). Detection of tyrosine with an excitation/emission of 280/305 nm (following quenching using EDTA) showed that the formation of DiY was matched by a decrease in tyrosine fluorescence in both the Aβ and vAβ MCO reactions compared with the samples incubated with CuCl_2_ alone or buffer only (Figure 1B). DiY fluorescence intensity continued to increase for the MCO reactions up to 2 h but did not increase further after 5 days (Figure 1C). However, by 5 days, small peaks could be observed for samples incubated with CuCl_2_ alone (Figure 1D), although the DiY signal remained negligible for buffer-only samples after 5 days incubation in the dark. Standard curve estimation of DiY content (Figure S1) revealed that 2 h MCO of Aβ and vAβ induced about 5 μM and ~12 μM DiY, respectively. As each DiY is contributed to by two molecules of Aβ, the percentage of Aβ molecules where Tyr is cross-linked is approximately 20% for Aβ and 48% for vAβ. Incubation of Aβ with CuCl_2_ alone for 2 h induced ~1 μM DiY, which further increased to ~2 μM at 5 days, which equates to approximately 4% of Aβ molecules participating in cross-links. Aβ and vAβ samples incubated with CuCl_2_ showed a decrease in tyrosine fluorescence after only 5 min (Figure 1B), indicating that the CuCl_2_ rapidly induces conformational changes (Roberts et al., 2012) in both Aβ and vAβ. This appears to be independent of DiY cross-linking, which only starts appearing ~2 h post-incubation. Overall, this suggests that the incubation of both Aβ and vAβ with CuCl_2_ alone or in combination with H_2_O_2_ results in the formation of DiY cross-links. DiY formation in the different samples occurs after different lengths of time depending on the oxidation conditions, which may impact Aβ assembly properties.

### Metal-Catalyzed Oxidation Influences the Assembly of Wild-Type Aβ, but Does Not Affect the Structure and Aggregation Propensity of Variant Aβ

**Figure 2:**
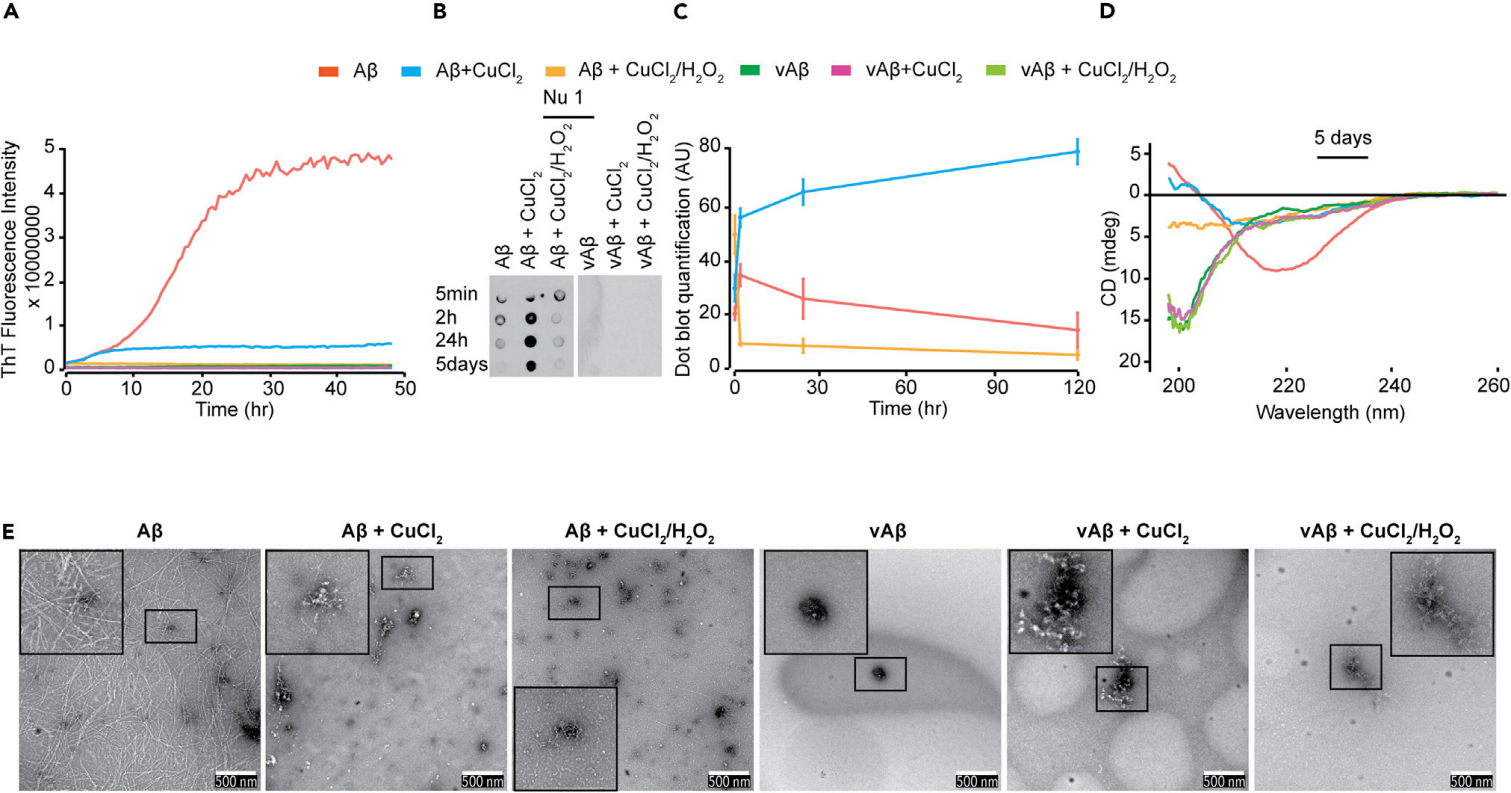
Metal-Catalyzed Oxidation Results in the Formation of Stabilized Aβ Assemblies (A–E) Th-T fluorescence was monitored for the freshly prepared Aβ and vAβ (50 μM) incubated at 37°C, without CuCl_2_, with CuCl_2_ (400 μM), or CuCl_2_ in combination with H_2_O_2_ (A). Dot blotting using NU-1 antibody identified Aβ oligomers in the Aβ samples, but not in vAβ reactions (B). Quantification of dot blotting signal over time reveals that the oligomers in the CuCl_2_-oxidized Aβ remain stable for 5 days, unlike in the other reactions in which very low NU-1 affinity signal was observed (C). CD at 5 days showed a high β-sheet content in the unoxidized Aβ sample, with reduced signal for CuCl_2_ Aβ, and CuCl_2_/H_2_O_2_Aβ. All vAβ samples showed spectra consistent with random coil conformation (D). TEM imaging at 5 days revealed a network of fibers in the unoxidized Aβ, whereas the CuCl_2_ Aβ showed clumped assemblies with very little fiber density and the CuCl_2_/H_2_O_2_ sample revealed amorphous-like assemblies. Unoxidized vAβ showed no assemblies, whereas both oxidized vAβ reactions showed amorphous-like aggregates (E). A minimum of three independent experiments was repeated to ensure the reproducibility of the findings. Scale bars, 500 nm. Error bars are expressed as ±SEM.

We have previously shown that DiY forms in both Aβ42 oligomers and fibrils (Al-Hilaly et al., 2013). Thioflavin T (Th-T) fluorescence assay was used to investigate whether DiY formation correlates with changes in the Aβ assembly (see Transparent Methods). As expected, the assembly-incompetent vAβ incubated in buffer shows no increase in Th-T fluorescence with time (Marshall et al., 2016). Wild-type Aβ gave the expected Th-T spectra showing a sigmoidal curve (Figure 2A) and the Th-T fluorescence increased further when Aβ was assembled in the presence of the metal chelator EDTA, which suggests some impact of trace metals on assembly properties (Figure S2). However, the Aβ and vAβ MCO reactions showed no Th-T fluorescence signal increase over the time frame of 50 h, indicating that either no self-assembly had taken place or that the assembly is significantly slow enough that the threshold of Th-T detection had not been reached. Wild-type Aβ incubated with CuCl_2_ shows a small fluorescence signal for DiY at ~ 2 h (Figure 1C) and Th-T spectrum showed a short lag phase (approximately up to 2 h) followed by a plateau at a low fluorescence signal. This appears to suggest that the Aβ assemblies formed become stabilized without further elongation (Figure 2A). vAβ incubated with CuCl_2_ showed no increase in Th-T fluorescence, consistent with the absence of assembly under these conditions.

We have previously shown that our Aβ preparation method results in the generation of monomers that assemble into oligomers detected by the oligomer-specific antibody, NU-1 (Lambert et al., 2007), before forming fibrils and amyloid plaques (Marshall et al., 2016) (see Transparent Methods). As expected, dot blotting with the NU-1 antibody revealed the presence of oligomers at 2 h in buffer-incubated Aβ samples, which disappeared over time (Figure 2B). In contrast, the buffer-incubated vAβ reactions showed no NU-1 reactivity, indicating the absence of oligomer formation as previously reported (Figure 1F) (Marshall et al., 2016). Aβ oligomers were only minimally detected in the Aβ MCO reaction at 5 min, which disappeared over time (Figures 2B and 2C). No NU-1 reactivity was observed for vAβ MCO reaction at any of the time points measured. Interestingly, the Aβ incubated with CuCl_2_ formed more oligomers early on, which persisted throughout the time studied. The data are consistent with the possibility that slower oxidation by CuCl_2_ facilitates Aβ oligomer formation and stabilization. The formation of DiY cross-linking correlates with this time point when Aβ is found in a NU-1 affinity conformation (Figures 2B and 2C).

Circular dichroism (CD) and transmission electron microscopy (TEM) were used to study the secondary structure and apparent morphologies of the resulting assemblies after 5 days incubation (see Transparent Methods). Spectra from vAβ under all three conditions showed a trough at 198 nm consistent with random coil conformation. Aβ in buffer showed a minimum at 218 nm consistent with an expected β-sheet conformation, typical of fibrillar Aβ (Figure 2D). Aβ CuCl_2_ shows a broad but weak minimum at 218 nm, but the signal from Aβ MCO reaction is too weak for assignment of secondary structure. This may be consistent with loss of protein from solution, which may arise from the formation of small, amorphous oligomers.

Electron micrographs showed an extensive fibril network for buffer-incubated Aβ, whereas CuCl_2_-oxidized Aβ showed clumped assemblies with a significantly reduced fiber density, and the MCO Aβ sample revealed amorphous structures with very few or no fibers (Figure 2E). Buffer-incubated vAβ sample showed very few assemblies of any kind. The vAβ MCO and CuCl_2_ reactions exhibited a few amorphous-like clumped assemblies. The CD and TEM data suggest that the MCO-catalyzed oxidation of Aβ prevents the formation of β-sheet-rich amyloid fibrils, whereas CuCl_2_-catalyzed oxidation generates oligomeric Aβ assemblies with some β-sheet conformation. Taken together, the results from Th-T, dot blotting, CD, and TEM revealed that the rapid DiY cross-linking of Aβ induced through MCO correlates with the association and trapping of Aβ as intermediates and inhibits their further assembly, whereas CuCl_2_ facilitates the formation and DiY cross-linking of Aβ oligomers into a long-lived oligomer population. In contrast, none of the conditions induced any assembly of vAβ into amyloid fibrils. Instead, the MCO and CuCl_2_ reactions resulted in association and trapping of vAβ into amorphous assemblies that show no evidence of β-sheet conformation.

### Photo-oxidation Induces Dityrosine Cross-Linking in Wild-Type Aβ and vAβ Peptides

**Figure 3:**
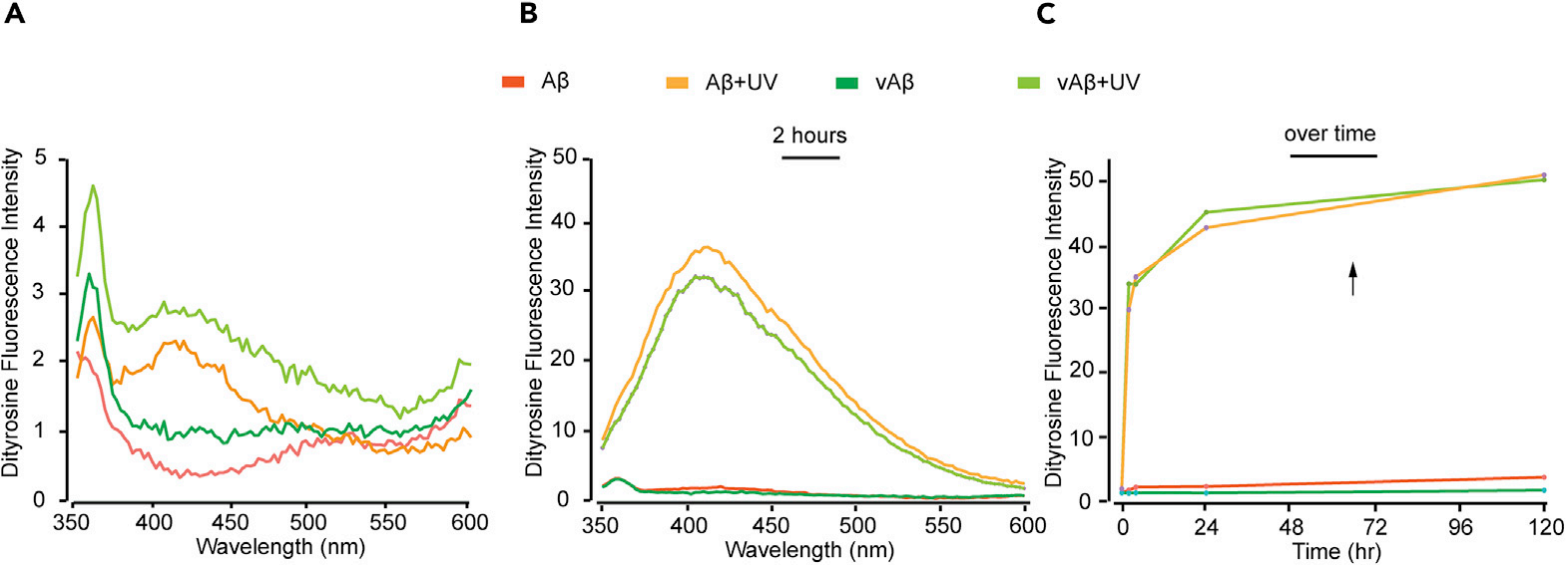
DiY Formation in Aβ and vAβ via UV Photo-oxidation (A–C) Freshly prepared Aβ and vAβ (50 μM) were incubated under UV. Fluorescence spectra were collected 5 min post-incubation using fluorescent excitation wavelength of 320 nm and emission collected between 340 and 600 nm, with DiY peak signal observed between 400 and 420 nm after 5 min of incubation (A), which increased following 2 h of incubation (B). Fluorescence intensity at 410 nm against time showed that incubation of the 2 h UV-exposed Aβ and vAβ samples in the dark resulted in further increase in DiY formation in the absence of the UV (C). The Aβ and vAβ samples that were not exposed to UV showed no DiY signal. A minimum of three independent experiments was repeated to ensure the reproducibility of the findings.

The aforementioned results suggest that copper contributes to DiY formation. However, previous studies have suggested that metals influence the assembly of Aβ (Barritt and Viles, 2015; Gu et al., 2018; Smith et al., 2006, 2007), and this is supported by the increased assembly of Aβ in the presence of EDTA (Figure S2). Therefore, to avoid the complication of using metals, which may influence assembly, UV photo-oxidation was used to oxidize Aβ and vAβ (Figure 3) (see Transparent Methods). UV oxidation of amino acids is limited to direct damage to Trp, Tyr, His, Cys Figure 7and cystine, but Met can be oxidized indirectly, usually in the presence of a sensitizer (Pattison et al., 2012). As before, the measurement of DiY formation was used to follow oxidation. Fluorescence spectra showed a small peak at 410 nm for both Aβ and vAβ after only 5 min incubation in UV. However, the intensity was lower than under MCO conditions (Figure 3A). After 2 h of UV exposure, a significant increase in intensity at 410 nm was observed (Figure 3B). Calculation of DiY content using the standard curve shown in Figure S1 suggested that approximately 12% of Aβ and 14% of vAβ molecules were involved in DiY, which is lower than following MCO treatment. After 2 h of UV exposure, samples were stored in the dark but despite this the intensity continued to increase for 120 h following incubation (18% DiY) (Figure 3C). These results show that UV photo-oxidation can induce DiY cross-linking for Aβ and vAβ peptides. The results are compared with peptides incubated without UV exposure for reference. Oxidation of other residues by the UV irradiation can not be ruled out, but here we focus on DiY to monitor the level of oxidation.

### Photo-oxidization Influences the Assembly of Wild-Type Aβ, but Does Not Impact on the Structure and Assembly of vAβ

**Figure 4:**
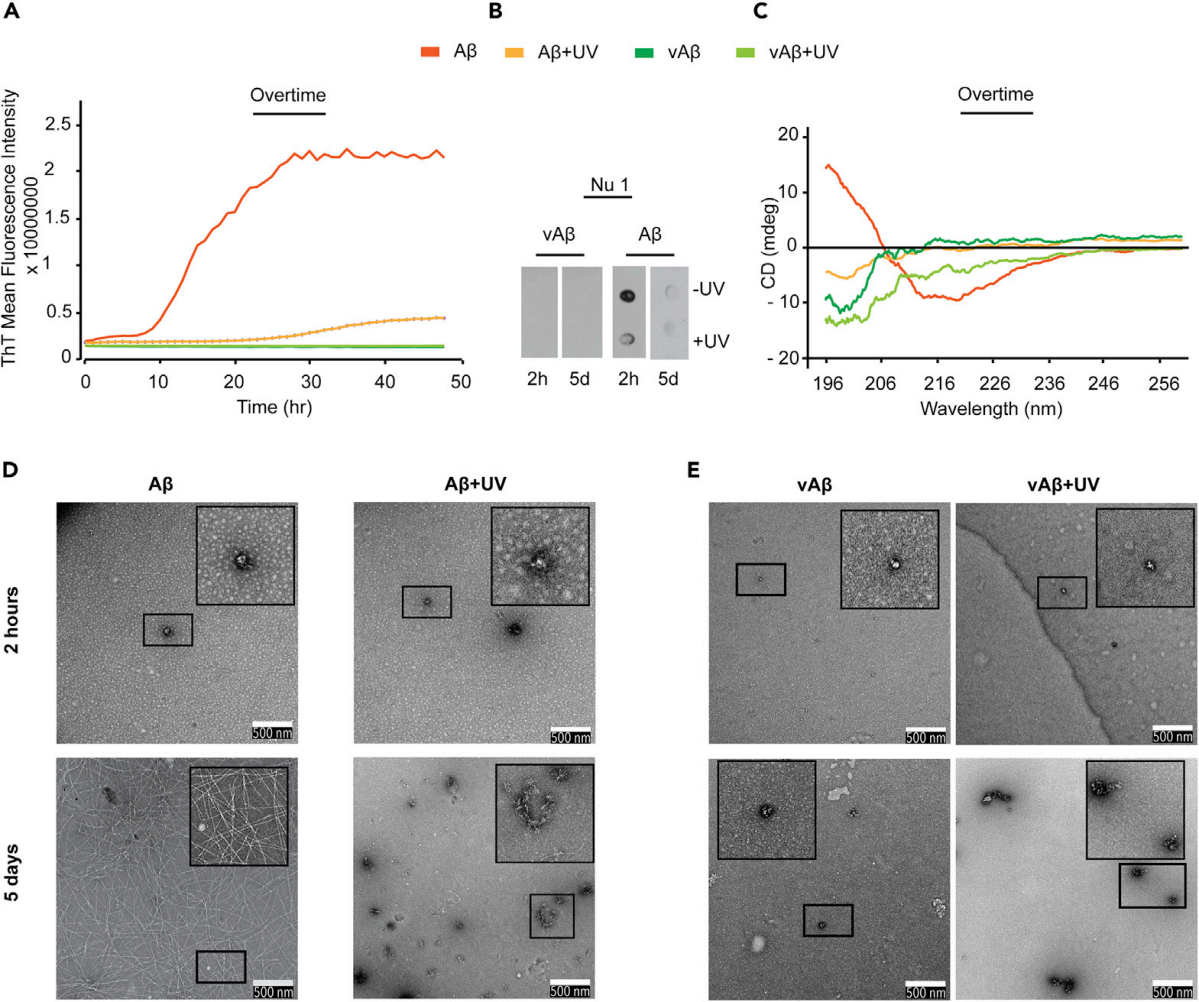
UV-Induced DiY Cross-Linking of Early Aβ Assemblies Correlates with Formation of Stabilized Assemblies (A) Th-T fluorescence spectrum shows the expected increase in fluorescence for assembling Aβ, but Aβ+UV Th-T fluorescence was significantly reduced. vAβ incubated in the absence or presence UV showed no Th-T fluorescence. (B) Dot blotting using NU-1 antibody shows binding suggesting fewer oligomers in the oxidized Aβ+UV than in the unoxidized Aβ sample. No binding of NU-1 was observed for Aβ+UV 5 days post-UV exposure, but a small signal was detected in the Aβ-UV sample. (C) CD at 5 days showed a high β-sheet content in the Aβ sample, whereas the oxidized Aβ+UV showed a loss of signal but indicated some random coil. Oxidized and unoxidized vAβ samples showed random coil conformation. (D) TEM after 2 h and 5 days showed that the unoxidized Aβ at 2 h formed oligomers, which transformed into a network of fibers at 5 days. The oxidized Aβ+UV samples formed small assemblies at 2 h, some of which developed into amorphous-like assemblies at 5 days. (E) vAβ does not assemble into amyloid fibrils, but vAβ+UV forms some amorphous aggregates after 5 days. A minimum of three independent experiments was repeated to ensure the reproducibility of the findings. Scale bars, 500 nm.

Th-T fluorescence was used to monitor the assembly of Aβ and vAβ following UV photo-oxidation for 2 h. The results are compared with peptides incubated without UV exposure for reference. A small increase in Th-T fluorescence was observed for Aβ+UV after a lag phase of 25 h, which may represent a slow assembly of the Aβ assemblies that escaped the less-efficient UV oxidation, whereas vAβ + UV showed no fluorescence intensity (Figure 4A). NU-1 dot blots showed no reactivity to vAβ + UV (Figure 4B). The Aβ+UV sample showed lower level NU-1 reactivity at 2 h compared with the Aβ control (Figure 4B), and reactivity disappeared after 5 days post-UV incubation in the cross-linked Aβ+UV sample, even though a small quantity of oligomers could still be detected in the control Aβ sample (Figure 4B). CD showed spectra consistent with random coil conformation for both vAβ and Aβ incubated under UV and a loss of signal that was more evident for Aβ than for vAβ (Figure 4C). By TEM, Aβ+UV and vAβ+UV again showed small assemblies at 2 h, which are still present alongside occasionally clumped amorphous-like assemblies after 5 days (Figures 4D and 4E). Similar to the results from MCO (Figure 2B), this suggests that the UV-induced DiY cross-linking correlates with the stabilization of Aβ and vAβ in a trapped, assembly-incompetent species. Other oxidation reactions may also be involved.

### Co-incubation with Oxidized Aβ Slows the Assembly of Freshly Prepared Aβ

**Figure 5:**
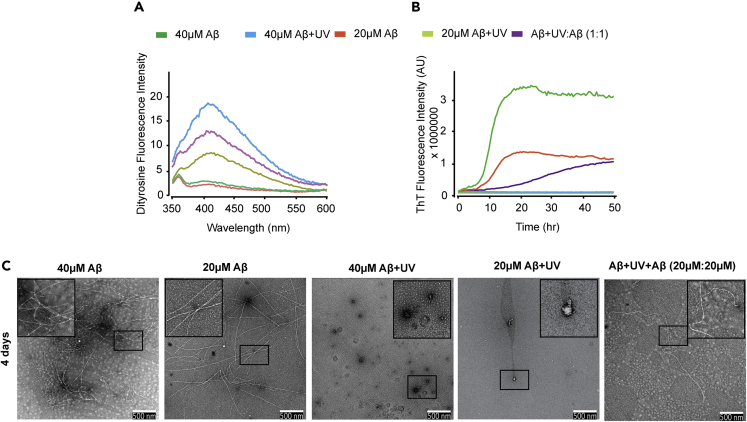
Co-incubation with Photo-oxidized, DiY-Cross-Linked Aβ Assemblies Slows the Aggregation of Freshly Prepared Aβ (A) DiY fluorescence was measured for freshly prepared Aβ (40 and 20 μM) and Aβ+UV:Aβ (20 μM: 20 μM) mixture immediately after co-incubation of freshly prepared Aβ with DiY-cross-linked Aβ. DiY signal was not detected in unoxidized freshly prepared 40 and 20 μM Aβ but was induced in 2 h UV-oxidized (Aβ+UV) samples and in the mixture. (B) Th-T fluorescence showed that the unoxidized 40 and 20 μM Aβ assemble at different rates, which was significantly delayed for Aβ+UV:Aβ-UV mixture and completely absent in the oxidized 40 and 20 μM Aβ samples. (C) TEM imaging at 4 days revealed the presence of fibrils in both unoxidized 40 and 20 μM Aβ, which was significantly reduced in the Aβ+UV:Aβ-UV mixture and absent in the oxidized 40 and 20 μM Aβ samples. A minimum of three independent experiments was repeated to ensure the reproducibility of the findings. Scale bars, 500 nm.

Our findings thus far suggest that oxidation results in the formation of DiY cross-links and results in stabilized Aβ assemblies that are prevented from further elongation into amyloid fibrils. To investigate this further, DiY cross-linking was induced in Aβ using 2 h UV exposure, and the sample was then incubated with an equal concentration of freshly prepared Aβ (20 μM:20 μM) and compared with a 40 μM Aβ sample not exposed to UV (see Transparent Methods for further detail). Fluorescence spectroscopy confirms the presence of DiY in the 40 μM Aβ sample exposed to UV, and the signal reduced by half when the oxidized 40 μM Aβ was diluted to 20 μM (Figure 5A); 20 μM oxidized Aβ added to 20 μM freshly prepared unoxidized Aβ (Aβ+UV:Aβ) revealed the presence of DiY signal (Figure 5A). Interestingly, the levels of DiY in the Aβ+UV:Aβ mixture was higher than that of 20 μM oxidized Aβ, suggesting that the presence of the UV-incubated Aβ in the environment results in the cross-linking of the freshly prepared Aβ following the co-incubation. Th-T fluorescence showed that the 40 μM Aβ sample showed a shorter lag phase and higher Th-T signal compared with the 20 μM Aβ sample confirming the expected concentration-dependent effect. The 20 μM and 40 μM oxidized Aβ samples showed no Th-T fluorescence intensity, similar to previous observations. When the Aβ mixtures were incubated together (Aβ+UV:Aβ 1:1), the mixture showed a longer lag phase (+20 h) but reached a similar Th-T intensity signal to the 20 μM unoxidized sample at 50 h. However, this was very significantly lower than the 40 μM unoxidized Aβ sample (Figure 5B). This suggests inhibition of self-assembly in the Aβ+UV:Aβ-UV mixture. At 4 days, TEM imaging revealed the presence of Aβ fibrils in both 20 and 40 μM Aβ samples, which were not detected in the photo-oxidized samples (Figure 5C). Only scant fibrils and smaller Aβ assemblies could be detected in the Aβ+UV:Aβ mixture, further confirming a reduced assembly in this mixture (Figure 5C). Taken together, this suggests that the incubation of freshly prepared Aβ with photo-oxidized Aβ results in an increase in DiY cross-linking and leads to the stabilization of some Aβ assemblies, resulting in the slower aggregation kinetics observed and prevention of elongation. DiY-containing, oxidized Aβ species may bind to Aβ and inhibit further elongation.

### Photo-oxidation of Pre-formed Aβ Assemblies Slows Further Assembly of Aβ Oligomers/Protofibrils and Prolongs Their Half-Life

**Figure 6:**
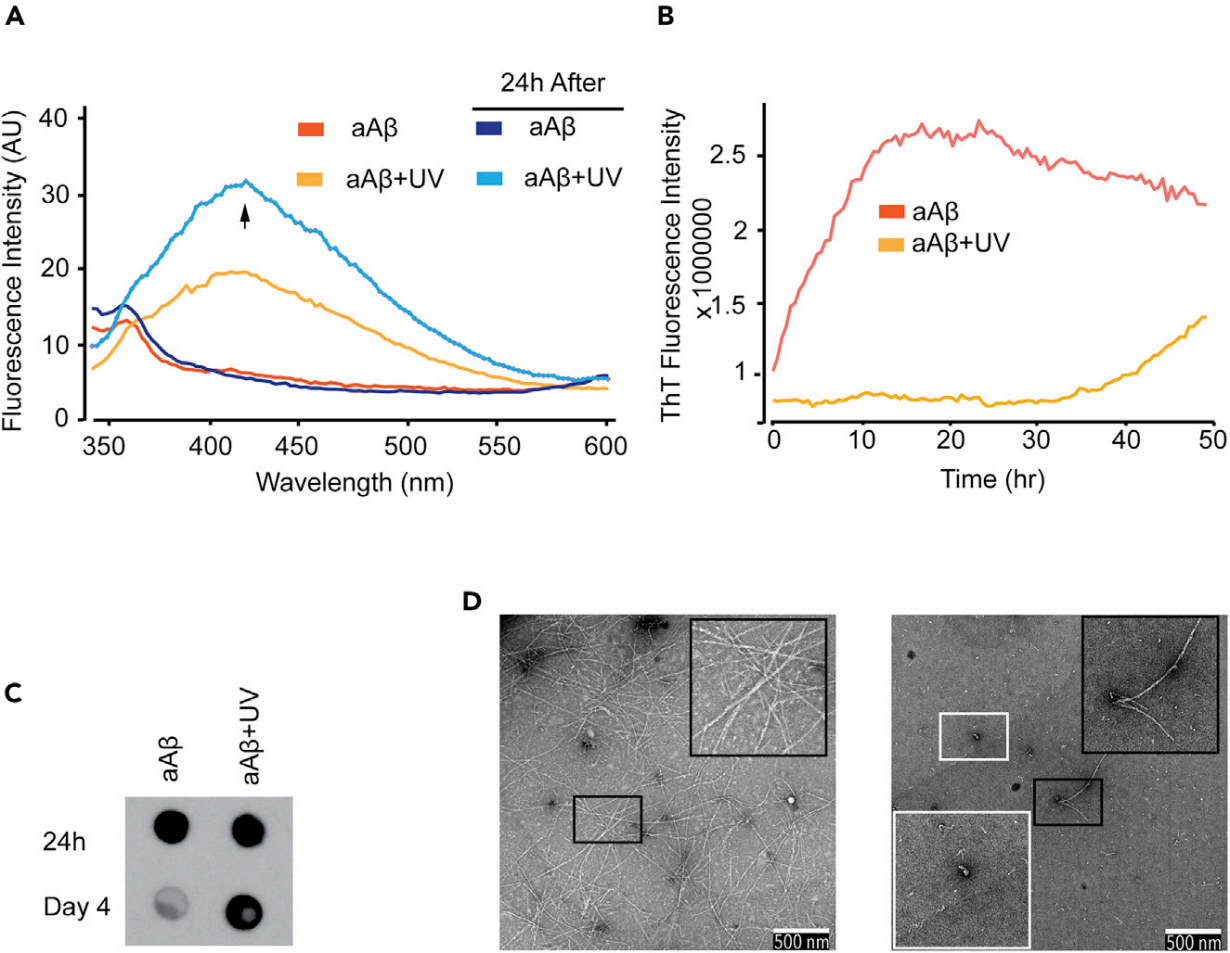
Photo-oxidation of Pre-formed Aβ (aAβ) (24 h) Assemblies Slows Further Assembly of Aβ Oligomers/Protofibrils and Prolongs the Half-Life of the Oligomers (A) DiY signal was detected in the oxidized aAβ+UV sample, which continued over time. Unoxidized aAβ samples showed no DiY signal. (B) Th-T fluorescence showed that the unoxidized aAβ continues to assemble, whereas the oxidized aAβ+UV became significantly inhibited from further elongation up to 40 h. (C) Dot blotting using NU-1 antibody reveals the presence of oligomers in the unoxidized and oxidized aAβ at the starting time point. However, the oligomers are still strongly detected in the oxidized aAβ+UV at 4 days, unlike in the unoxidized samples. (D) TEM imaging at 4 days revealed the presence of fibrils in the unoxidized aAβ sample, whereas the oxidized aAβ+UV showed oligomers and a reduced number of fibrils. A minimum of three independent experiments was repeated to ensure the reproducibility of the findings. Scale bars, 500 nm

Our findings thus far strongly suggest that oxidation stabilizes and strongly slows the aggregation and further elongation of Aβ assemblies. However, it is not clear whether these could also alter the assembly of preformed Aβ assemblies. To investigate this, Aβ (50 μM) was freshly prepared and allowed to assemble for 24 h (henceforth called aAβ). aAβ samples were exposed for 2 h to UV to induce DiY cross-linking (aAβ+UV) (Figure 6) (see Transparent Methods). Unlike samples that were not exposed to UV (aAβ), the aAβ+UV samples showed an increasing intensity arising from DiY, which continued to increase even after incubation in the dark following UV exposure (Figure 6A). Th-T fluorescence intensity showed that aAβ continues to assemble, reaching plateau after approximately 15 h (Figure 6B). However, DiY cross-linking induced by UV correlates with the inhibition of further assembly of the aAβ+UV for ~40 h, suggesting that the photo-oxidation leads to the stabilization or trapping of aAβ+UV (Figure 6B). A gradual increase in fluorescence is observed beyond 40 h, which may indicate delayed assembly. Dot blotting using NU-1 revealed a similar level of oligomers in the oxidized and unoxidized aAβ samples at the starting point (Figure 6C). Interestingly, the oligomers in the oxidized aAβ+UV sample persisted beyond 4 days, unlike the unoxidized aAβ sample, which showed a very low level of oligomers at the later time point (Figure 6C). TEM imaging showed the presence of oligomeric assemblies and scant fibers in the oxidized aAβ+UV sample, compared with the unoxidized aAβ, which showed extensive fibril network. Together, these data provide further evidence indicating that photo-oxidation of pre-formed Aβ fibrils leads to the formation of DiY cross-links and results in stabilization of aAβ assemblies, which prevent or delay further elongation. This is similar to the stabilization of Aβ oligomers observed following the slower/milder DiY cross-linking in the Aβ/CuCl_2_ preparation, which occurred after the formation of Aβ oligomers (Figures 2B and 2C).

### Self-Assembly Is Important for Aβ Toxicity

**Figure 7:**
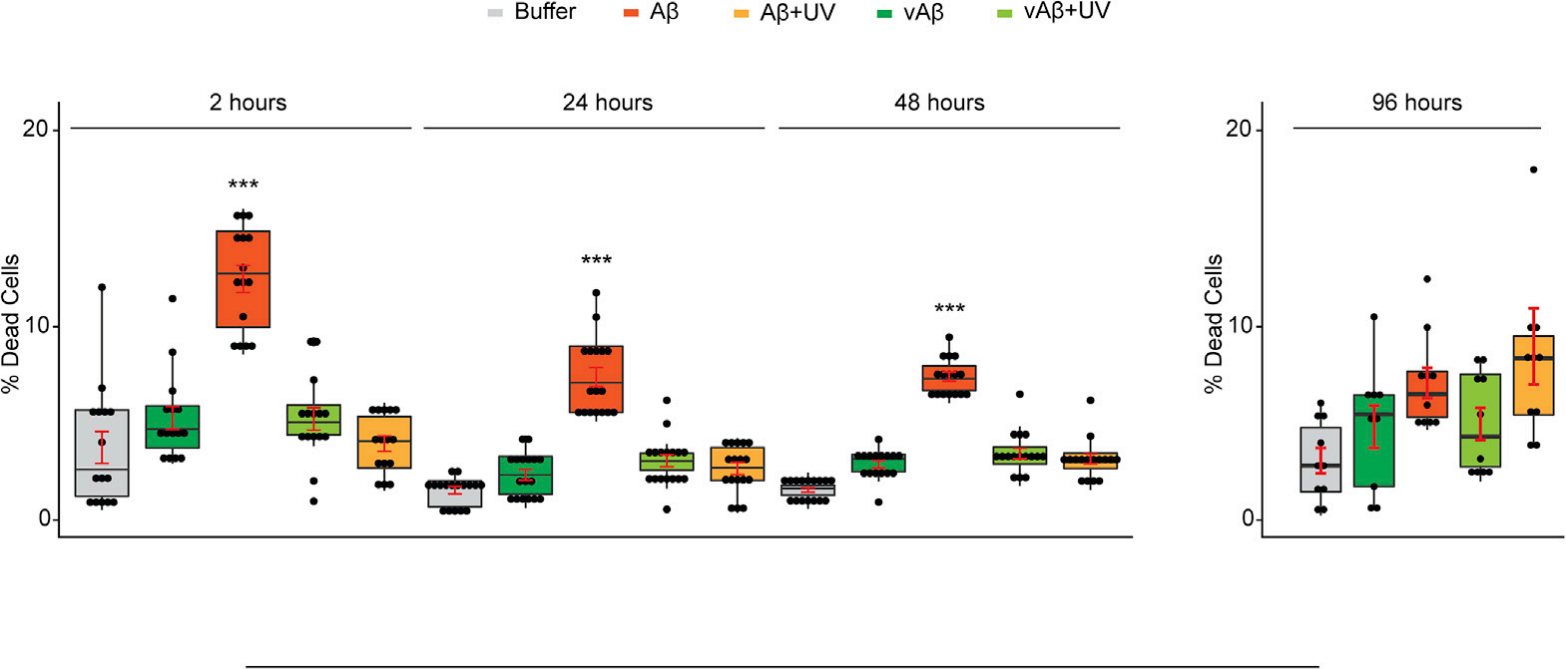
Oxidized, DiY-Containing Aβ Assemblies Are Not Toxic to Cells Differentiated SH-SY5Y neuroblastoma cells were incubated with UV oxidized or unoxidized vAβ or Aβ for 3 days, following which the percentage of dead cells was quantified using ReadyProbes Live/Dead Assay. The Aβ and vAβ samples were freshly prepared and UV oxidized for 2 h (Aβ+UV/vAβ+UV), or UV oxidized for 2 h followed by a further incubation on bench and in the dark for 24, 48, or 96 h, before being administered to cells. vAβ and Aβ samples not exposed to UV were used as reference. Only the unoxidized/assembling wild-type Aβ induced significant cell death. Experiments were repeated five times. ∗∗∗p < 0.001. p ≤  0.05 (∗), <0.01 (∗∗), <0.0001 (∗∗∗∗) and >0.05 was not significant. Error bars are expressed as ±SEM.

Multiple pieces of evidence have shown the detrimental role of Aβ on neuronal function that eventually leads to neuronal death (Marshall et al., 2016; Lambert et al., 1998; Lacor et al., 2007; Zhang et al., 2014; Selkoe and Hardy, 2016). Our Th-T, dot blot, and TEM data showed that Aβ42 monomers self-assemble to form significant level of oligomers at 2 h, and eventually protofibrils, fibrils, and a network of fibrils at 4 days (Figures 2 and 6). Hence, the rate of self-assembly is high at early time points and plateaus at later time points when Aβ forms fibril network and plaques (Marshall et al., 2016). To investigate the role of self-assembly in toxicity, ReadyProbes live/dead assay was conducted on terminally differentiated SH-SY5Y neuroblastoma cells incubated for 3 days with wild-type Aβ42 prepared in the following manner; Aβ42 was allowed to assemble for 2, 24, 48, and 96 h to form oligomers, protofibril mixture, and fibrils and then treated for 2 h with UV (Aβ+UV) to induce DiY, or untreated (Aβ) (see Transparent Methods). vAβ was used as an assembly-incompetent control (Marshall et al., 2016). Our results reveal a significantly higher level of cell death in wells incubated with Aβ after 2 h of preparation, compared with cells treated with Aβ after 24, 48, and 96 h incubation consistent with previous observations (Figure 7) (Marshall et al., 2016). However, the none of the oxidized Aβ samples containing DiY showed any toxic effect on the cells, similar to the assembly-incompetent oxidized and unoxidized vAβ (Figure 7). This suggests a key role for self-assembly in the toxicity of Aβ.

## Discussion

In AD, Aβ self-assembles to dimers, oligomers, fibrils, and eventually amyloid plaques, one of the key hallmarks of the disease. Aβ is generally accepted to play a key role in AD, but the mechanism behind its toxicity is still not completely understood. Numerous studies have searched for the elusive “toxic” species and attempted to characterize its structure, and this has identified supposedly toxic assemblies such as dimers, 12mers, ∗56 KDa and hexamers (Lesne et al., 2006; Reed et al., 2011; Benilova et al., 2012). Here, we show that oxidation results in the formation of DiY cross-links which is one of several possible oxidative modifications and this significantly slows, or halts the self-assembly of Aβ42, trapping it in a specific state. We compared the self-assembly and toxicity of assembly-incompetent vAβ with wild-type Aβ and oxidized, trapped Aβ.

MCO and photo-induced oxidation of vAβ revealed that DiY forms very rapidly as early as 5 min post-oxidation. However, oxidation did not lead to vAβ assembly even after 5 days in the oxidative environment or post-oxidation. This demonstrates that the oxidation and the formation of DiY does not induce aggregation of the vAβ, which is known not to self-assemble (Marshall et al., 2016). DiY was also rapidly induced in wild-type Aβ using both MCO and photo-induced oxidation; however, further assembly is inhibited or significantly slowed. Co-incubation of DiY-containing Aβ with freshly prepared uncross-linked Aβ demonstrated significantly reduced assembly. This suggests that oxidation and DiY cross-linking does not induce or facilitate the aggregation of the wild-type Aβ. Instead, Aβ assemblies are trapped and further elongation is delayed. This is supported by previous reports that showed that DiY-cross-linked Aβ are slow to fibrilize and form long-lived soluble oligomeric aggregates (Kok et al., 2013; O'malley et al., 2014, 2016; Sitkiewicz et al., 2014). Mass spectrometry studies have revealed that DiY cross-linking leads to the stabilization of Aβ40 in compact oligomeric species (Sitkiewicz et al., 2014), which is in strong support of our findings.

Previous studies have suggested that DiY cross-linking can facilitate Aβ assembly (Atwood et al., 2004; Yoburn et al., 2003; Barnham et al., 2003; Zhang et al., 2017) or inhibit or slow Aβ self-assembly (Smith et al., 2007; Gu et al., 2018) or stabilize assemblies (Vazquez et al., 2019). Importantly, it has been shown that copper influences self-assembly in different ways depending on the concentration and ratio (Matheou et al., 2015; Sarell et al., 2010), which may go some way to explaining these inconsistencies. These discrepancies could also arise from the different methods used to induce DiY in Aβ. Previous studies have used varying concentration of horseradish peroxidase and H_2_O_2_ (Galeazzi et al., 1999; Yoburn et al., 2003; Ali et al., 2006; Sitkiewicz et al., 2014; O'malley et al., 2014), varying concentrations of copper and H_2_O_2_ (Yoburn et al., 2003; Atwood et al., 2004; Barnham et al., 2004; Smith et al., 2007; Davis et al., 2011; Al-Hilaly et al., 2013; Williams et al., 2016), copper in combination with ascorbate (Gu et al., 2018), or photo-oxidation (Yoburn et al., 2003; Williams et al., 2016) to induce DiY cross-links in Aβ. In addition, even small differences in incubation conditions (e.g., trace metals, temperature, etc.) as well as atmospheric ozone levels (Vazquez et al., 2019) would result in different levels of DiY cross-links formed, which may result in varying impact on Aβ assembly. Moreover, the nature of the DiY cross-links may differ from one protocol to another. For example, other amino acids, such as phenylalanine, could modulate formation of cross-links (Zhang et al., 2019). Methodologies are likely to induce different oxidative effects on other amino acid side chains such as histidine, lysine, and met35 of Aβ (Kowalik-Jankowska et al., 2004; Ali et al., 2005; Palmblad et al., 2002; Friedemann et al., 2015). For example, met35 oxidation has been shown to attenuate Aβ oligomer formation and to enhance oxidation of Aβ Y10 (Palmblad et al., 2002). Mapping the specific modifications induced by the oxidation methods in a time-dependent manner using mass spectrometry would help to provide clarity on the relative role for DiY on Aβ assembly and whether this occurs in combination with other modifications to Aβ. This is a part of further studies.

Furthermore, the method of Aβ preparation used before DiY cross-linking may be critical in the outcome of the DiY cross-links on Aβ. Different sources and methods are used to prepare Aβ, resulting in a diverse pool of Aβ aggregates (Benilova et al., 2012). Aβ exists in a pool of monomers, soluble oligomers, and insoluble fibrils. Multiple studies have reported that the soluble Aβ oligomers in AD are composed of dimers, trimers, tetramers, pentamers, and decamers; Aβ-derived diffusible ligands (ADDLs); dodecamers; and Aβ∗56 (Benilova et al., 2012). A question therefore arises regarding how DiY cross-linking impacts these pools of assemblies: presumably by creating new cross-linked dimeric species. Previous molecular dynamics studies have revealed that induction of DiY in a pool of monomeric Aβ42 results in conformationally altered dimers that expose hydrophobic residues that may be limited to forming trimers via hydrophobic rather than polar interactions (Zhang et al., 2017). Our previous data showed that within 2 h of preparation, Aβ exists mostly as oligomers with a random coil conformation with a small β-sheet contribution (Marshall et al., 2016). Here, our results showed that the very slow oxidation induced by CuCl_2_ alone first facilitates the formation of Aβ oligomers followed by DiY cross-linking of the oligomers resulting in a stabilized oligomeric population. The more rapid MCO reaction results in DiY formation as early as 5 min post-oxidation whereby the Aβ becomes trapped in a pre-oligomeric conformation (as assessed using the antibody NU-1). Th-T fluorescence, CD, and TEM showed that photo-oxidation of early Aβ species traps Aβ in a random coil conformation and prevents or significantly delays further assembly into amyloid fibrils. UV oxidation and DiY cross-linking in preformed oligomer/protofibril assemblies similarly results in the stabilization of this state and significantly delays further elongation to fibrils. Taken together, these results suggest that the timing of oxidation of Aβ critically influences its assembly, leading to the stabilization or significantly reduced assembly of the Aβ assemblies, which correlates with the time of cross-linking.

Aβ self-assembly is believed to be important for its toxicity (Marshall et al., 2016), and many studies have implicated the role of oligomeric species in cytotoxic effects (Glabe and Kayed, 2006; Soura et al., 2012; Walsh et al., 1999). Here, we compared toxicity of the unoxidized Aβ with Aβ that had been photo-oxidized and DiY cross-linked *in vitro*, whereby specific species in the assembly pathway have been stabilized. Aβ42 was oxidized at different time points to stabilize a series of pre-oligomer, oligomeric, protofibrillar, and fibrillar forms. We show that none of these species is able to induce cell death following 3 days of incubation with differentiated neuroblastoma cells, whereas unoxidized, oligomeric Aβ remained potently toxic. This finding is in conflict with previous studies that showed that DiY Aβ42 assemblies are toxic to cells (Barnham et al., 2004); DiY-cross-linked Aβ40 dimers induce cell viability loss (Kok et al., 2013) and that Th-T positive, DiY-cross-linked Aβ40 fibrils were able to inhibit long-term potentiation (LTP) (O'malley et al., 2014). We also observed that oxidized vAβ is not toxic to cells, suggesting that the presence of DiY alone is not sufficient to induce toxicity. The oxidized and DiY-cross-linked Aβ42 assemblies produced here are different from DiY Aβ40 reported by others (O'malley et al., 2014; Kok et al., 2013), as the DiY Aβ42 produced shows little Th-T fluorescence intensity and does not proceed to form fibrils. However, we do not rule out the possibility that DiY Aβ preparation in our study and others also results in other oxidative modifications, which may explain the discrepancies between these studies.

It is important to note that method of peptide preparation, peptide type, peptide concentration and aggregation state, and model system used may play a huge role in determining cell toxicity (Jana et al., 2016; Kaniyappan et al., 2017; Cecchi et al., 2008; Krishtal et al., 2015). Previous studies on DiY Aβ toxicity have used LTP (O'malley et al., 2014), MTS proliferation assay (Barnham et al., 2004), MTT. and lactate dehydrogenase assay (Ono et al., 2009) with varying Aβ concentrations to determine DiY Aβ toxicity. These assays, although showing the presence of cell injury, do not quantify absolute cell death. Impaired spine morphology and density, accompanied by increased reactive oxygen species and intracellular calcium, without apparent cell death have been reported as a result of tau toxicity (Kaniyappan et al., 2017). We have shown that a very short, 2 h exposure to Aβ oligomers of differentiated SHSY5Y cells result in oxidative and nucleolar stress without DNA damage or neuronal death (Maina et al., 2018). Thus, some discrepancies may arise from the assays and the cell model used. Moreover, if DiY Aβ is toxic, then it may depend on the level of DiY. None of the previous studies that studied DiY Aβ toxicity quantified the level of DiY formed on Aβ. As a result, discrepancies may also arise from the differences in the quantity of DiY cross-links in the Aβ used in toxicity assays. Indeed, we observed different levels DiY intensity between MCO and UV treatments.

Nonetheless, here we show that oxidation of Aβ *in vitro* leads to formation of DiY, halts Aβ self-assembly, and prevents cytotoxicity in a live-dead assay. We have previously demonstrated that assembly-incompetent vAβ is not toxic to cells (Marshall et al., 2016). We therefore conclude that continued self-assembly is important for Aβ toxicity. We believe that the timing of the oxidation may be critical. For example, formation of DiY in Aβ fibrils would promote its stability and formation of amyloid plaques. Indeed, we have previously shown the presence of DiY on Aβ plaques in AD brain tissue and demonstrated that DiY Aβ fibrils become highly insoluble and resistant to formic acid denaturation (Al-Hilaly et al., 2013).

In conclusion, oxidation, which results in DiY cross-linking, promotes Aβ stabilization and does not induce or facilitate Aβ assembly. Our findings strongly suggest a role for self-assembly for Aβ toxicity. We show that Aβ exerts a high level of toxicity at a stage when self-assembly potential is high, compared with when the self-assembly rate is significantly diminished or abolished, as is the case for oxidized and vAβ. This is observed even for those preparations wherein oligomeric Aβ has been stabilized. Our work implies that the timing of DiY formation plays a key role in further assembly and stability of Aβ.

### Limitations of the Study

Here, we have provided evidence to show that oxidative conditions can induce the formation of DiY cross-links in Aβ42 using MCO and UV photo-oxidation *in vitro*. We show that oxidation under the conditions used here halts further assembly. Stabilized Aβ42 following oxidation is non-toxic to differentiated neuroblastoma cells. However, our study was not able to fully characterize whether other amino acids were affected by oxidation and what impact this might have on the prevention of assembly. We confirm that DiY is a major outcome of oxidation. Our work shows that oxidation of Aβ *in vitro* results in formation of non-toxic Aβ species. However, oxidative stress is known to be an important trigger for neurodegenerative diseases and our results do not imply a protective effect of oxidative stress. Oxidation has been performed under controlled environment *in vitro* affecting only Aβ self-assembly. Oxidation of Aβ *in vivo* is likely to have made diverse effects that have not been addressed in this study.

### Resource Availability

#### Lead Contact

Further information and requests for resources of reagents should be directed to and will be fulfilled by the Lead Contact, Louise Serpell l.c.serpell@sussex.ac.uk.

#### Materials Availability

This study did not generate new unique reagents.

#### Data and Code Availability

This study did not generate code. The published article contains all datasets generated or analyzed during this study.

## Methods

All methods can be found in the accompanying Transparent Methods supplemental file.

## Supplemental Information

Document S1. Transparent Methods and Figures S1 and S2
