# Supplementary material for: Metal- and UV- Catalyzed Oxidation Results in Trapped Amyloid-β Intermediates Revealing that Self-Assembly Is Required for Aβ-Induced Cytotoxicity
Source: iScience. 2020 Sep 6;23(10):101537. doi: 10.1016/j.isci.2020.101537 (PMC7516296; doi:10.1016/j.isci.2020.101537)
Supplement: Document S1. Transparent Methods and Figures S1 and S2 [file mmc1.pdf]

iScience, Volume *23*

Supplemental Information

Metal- and UV- Catalyzed Oxidation Results in Trapped Amyloid-β Intermediates Revealing that Self-Assembly Is Required for Aβ-Induced Cytotoxicity

Mahmoud B. Maina, Gunasekhar Burra, Youssra K. Al-Hilaly, Kurtis Mengham, Kate Fennell, and Louise C. Serpell

Supplemental information

Transparent methods

Materials and Methods

Preparation of Aβ

Recombinant Aβ42 (Aβ) and variant Aβ42 (vAβ) were purchased in Hexafluoroisopropanol (HFIP) films from rPeptide and JPT, respectively. vAβ is a variant of Aβ1-42 with F19S and G37D mutations which render the peptide assembly incompetent (see (Marshall et al. 2016) for detail). The peptides were prepared using an established protocol, and all procedures were done using protein LoBind Eppendorfs and tips. 0.2 mg/mL of the peptides were solubilized in 200 µL HFIP (Sigma-Aldrich), vortexed for 1 min and sonicated in 50/60 Hz bath sonicator for 5 min. The HFIP was removed by air drying using a low stream of nitrogen gas. The dried peptide films were dissolved in 200 µL Dimethyl sulfoxide (DMSO) >99.9% (Sigma-Aldrich), vortex for 1 min and sonicated for 1 min. The solution was passed through a 2 mL 7K MWCO Zeba buffer-exchange column (Thermo Scientific) and stacked with 40 µL of 10 mM phosphate buffer (pH 7.4). The concentration of the peptides was determined using a NanoDrop spectrophotometer (Thermo Scientific) at a wavelength of 280 nm (extinction coefficient of 1490 $M^{-1}cm^{-1}$). The peptide solutions were immediately diluted to 50 µM with the 10 mM phosphate buffer and used as indicated. For the experiments involving preformed Aβ assemblies, the 50 µM Aβ was left to assemble for 24 without shaking before being subjected to UV exposure.

Metal-catalysed oxidation (MCO) of Aβ and vAβ

Freshly prepared samples of Aβ1-42 and vAβ1-42 peptides (50 µM) in 10 mM phosphate buffer, pH7.4 were incubated i) without $CuCl_2$, ii) in the presence of 400 µM $CuCl_2$ (peptide: $CuCl_2$ ratio 1:8) and iii) in the presence of 400 µM $CuCl_2$ and 2.5 mM H_2O_2. At this concentration, we did not observe any precipitation of copper phosphate. An additional control was performed adding ethylenediaminetetraacetic acid (ETDA) (2 mM) to the assembly mixture of wild type Aβ. The peptides were incubated at 37°C without agitation and at each time point collected, the oxidation reaction was quenched using EDTA at a final concentration of 2 mM. A minimum of three independent experiments were conducted to ensure the reproducibility of the findings.

Standard curve of dityrosine

Dityrosine was synthesised as described in (Al-Hilaly *et al.*, 2013). To generate a dityrosine standard curve, a set of dityrosine standard concentrations (0.1, 0.5, 1, 2, 3, 5 and 10 µM)

were prepared in Milli-Q water. Each concentration was prepared in triplicate and dityrosine fluorescence was recorded for each concentration using excitation wavelength 280 nm and emission wavelength 410 nm. The mean values of dityrosine fluorescence intensity for each concentration were plotted against dityrosine concentration and line plot was constructed by linear regression analysis using Microsoft Excel software. The equation of this line was used to quantify the dityrosine content of Aβ samples oxidized with MCO or $CuCl_2$ alone.

Photo-oxidation of Aβ and vAβ

Freshly prepared samples of Aβ1-42 and vAβ1-42 peptides (50 μM) in 10 mM phosphate buffer, pH 7.4 were incubated i) without UV-C in the dark, and ii) under of UV-C for 5min or 2h using a G6T5 Germicidal 9′ 6W T5 UVC lamp set to 8 $J/m^2/sec$ (General Lamps Ltd). A minimum of three independent experiments were conducted to ensure the reproducibility of the findings.

Fluorescence spectroscopy

The formation of dityrosine was monitored with a fluorescence spectrophotometer (Varian Ltd., Oxford, UK), using a 1 cm path length quartz cuvette (Starna, Essex, UK). The presence of dityrosine was detected using fluorescent excitation wavelength of 320 nm and emission collected between 340 – 600 nm, with dityrosine peak signal observed between 400-420 nm. Tyrosine fluorescence signal was monitored using an excitation wavelength of 280 nm and an emission wavelength of 305 nm, with the peak tyrosine emission observed at 305 nm. For experiments involving metal-catalysed oxidation, the reaction was quenched using EDTA to a final concentration of 2 mM. For all the measurements, the excitation and emission slits were both set to 10 nm, scan rate set to 300 nm/min with 2.5 nm data intervals and an averaging time of 0.5 s. The photomultiplier tube detector voltage was set at 500 V.

Thioflavin T fluorescence assay of Aβ self-assembly

Samples were incubated with 100 μM Thioflavin T (Th-T), and the rate of Th-T binding was monitored over time at 37°C using SpectraMax i3 plate reader with samples incubated in CellCarrier-96 Ultra Microplates (PerkinElmer). The readings were collected in a black 96-well plate with a clear bottom (PerkinElmer), which was sealed with an optically clear polyolefin film to avoid evaporation (StarSeal Advanced Polyolefin Film, Starlab). The number of readings per well was set to 6, PMT voltage was set to high and blank spectra of the buffer were subtracted to protein fluorescence scans. The excitation wavelength was set at 440 nm, and emission at 483 nm and the signal collected every 30 min, with 5 sec low orbital shakes before readings. The fluorescence data were plotted against time. A minimum of three independent experiments was repeated to ensure the reproducibility of the findings.

Circular Dichroism (CD)

The secondary structure of Aβ and vAβ peptides at 50 μM concentration in 10 mM phosphate buffer (pH 7.4) incubated under different conditions was assessed using Jasco J715 CD spectrometer (Jasco, Goh-Umstadt, Germany). 140 μL of each sample was placed into a 1 mm path length quartz cuvette (Hellma) and scanned between 190 nm and 260 nm. The CD spectra were collected in triplicate at a maintained temperature of 21 °C.

Negative-stain transmission electron microscopy (TEM)

The morphology of control and cross-linked Aβ and vAβ peptides was assessed by negative stain TEM. Briefly, 4 μL of each sample was dropped onto 400-mesh carbon-coated grids (Agar Scientific, Essex, UK). After 1 min incubation, the excess sample was blotted using filter paper, and the grid was washed with 4 μL filtered Milli-Q water and blotted. The grid was then negatively stained for 40 sec using 4 μL of filtered 2% (w/v) uranyl acetate. The excess stain was blotted with filter paper and grids left to air-dry before storage. The grids were examined on a Jeol Jem1400-plus transmission electron microscope (Jeol, USA), operated at 80 kV fitted with a Gatan Orius SC100 camera (UK).

Dot-blotting

A total of 4 μl was spotted onto a 0.2 μM pore nitrocellulose membrane and allowed to dry for 10 min. The membrane was boiled with PBS for 1 min twice and then blocked with blocking buffer (5% milk in 0.05% TBS-T) for 1 hour at room temperature on a rocker. The blocking buffer was next replaced with mouse NU-1 primary antibody (1/2000) and left to bind overnight at 4°C on a rocker. The membrane was washed 6 times for 5 min with washing buffer (0.05% TBS-T), then incubated with an HRP-conjugated goat anti-mouse secondary antibody for 1 hour. The membrane was washed six times for 5 min with washing buffer, then incubated with Clarity Western ECL Substrate (Bio-Rad) for 1 min before being developed in the darkroom. The NU-1 antibody was a gift from the William Klein lab (Lambert et al.). A minimum of three independent experiments were conducted to ensure the reproducibility of the findings.

Cell death assay

Differentiated SHSY5Y neuroblastoma cells were used for the toxicity experiments. Firstly, undifferentiated SHSY5Y neuroblastoma cells (ATCC CRL-2266™), were maintained in Dulbecco's Modified Eagle Medium: Nutrient Mixture F-12 (DMEM/F-12) (Life Technologies, United Kingdom), supplemented with 1% (v/v) L-glutamate (L-Gln) (Invitrogen), 1% (v/v) penicillin/streptomycin (Pen/Strep) (Invitrogen) and 10% (v/v) Fetal Calf Serum at 37°C and 5% CO2. The undifferentiated SHSY5Y cells were seeded to 60% confluency in a CellCarrier-

96 Ultra Microplates (PerkinElmer). The cells were differentiated in a medium containing 1% Fetal Calf Serum supplemented with 10 µM trans-Retinoic acid (Abcam) for 5 days. Next, the medium was replaced with a serum-free media supplemented with 2 nM brain-derived neurotrophic factor (BDNF) (Merck Millipore). After 2 days in the BDNF-containing media, the media was replaced with serum-free media and the cells were treated with UV cross-linked or uncross-linked vAβ or Aβ for 3 days. At the end of the incubation period, the cells were incubated with ReadyProbes reagent (Life Technologies) for 15 min. The ReadyProbes kit contains NucBlue Live reagent that stains the nuclei of all live cells and Propidium iodide that stains the nuclei of dead cells with compromised plasma membrane. The cells were imaged at 37°C and 5% CO_2 using Operetta CLS high-content analysis system (PerkinElmer) using DAPI and TRITC filters. At least 5000 dead and live cells were analysed using the Harmony software automated analysis algorithm within the Operetta CLS high-content analysis system. A minimum of three independent experiments were repeated to ensure the reproducibility of the findings.

Supplemental Results
Figure S1

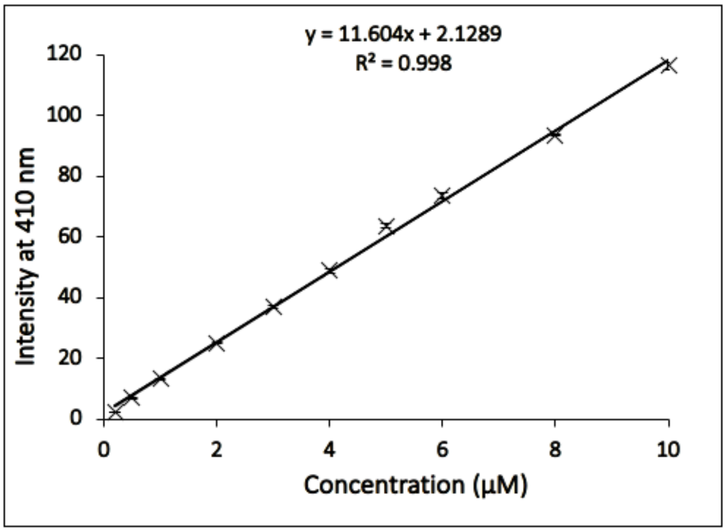

Figure S1. Related to Figure 1. Standard curve showing the concentration of DiY standard against intensity at 410 nm. Data was recorded as described in Methods above.

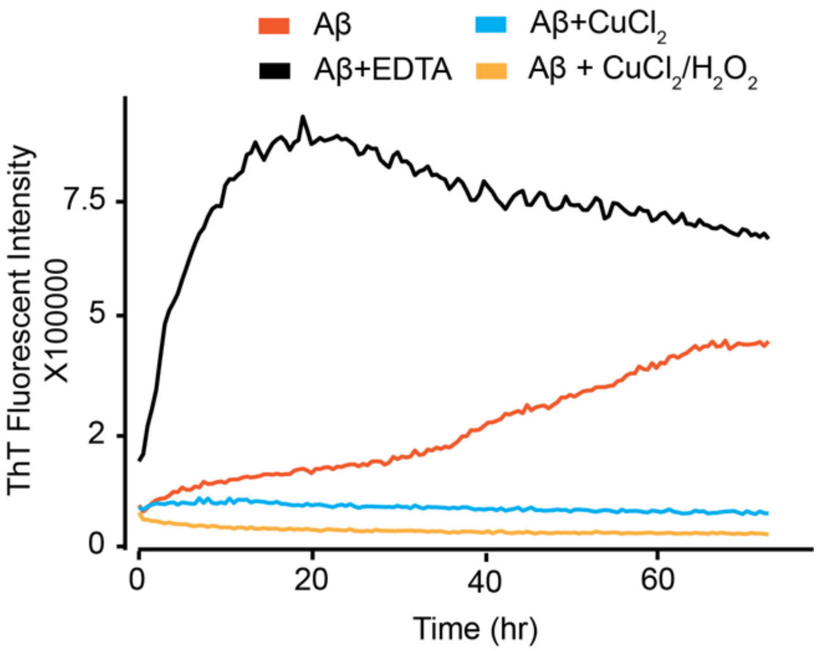

Figure S2. Related to Figure 1. Thioflavine T fluorescence assay comparing Aβ1-42 self-assembly under MCO conditions, copper alone and in buffer with EDTA to chelate trace metals. Aβ1-42 self-assembly at a higher rate than Aβ1-42 in buffer suggesting that the trace metals present in the water used to make the buffer can influence the assembly rate.

References

AL-HILALY, Y. K., WILLIAMS, T. L., STEWART-PARKER, M., FORD, L., SKARIA, E., COLE, M., BUCHER, W. G., MORRIS, K. L., SADA, A. A., THORPE, J. R. & SERPELL, L. C. 2013. A central role for dityrosine crosslinking of Amyloid-beta in Alzheimer's disease. *Acta Neuropathol Commun,* 1**,** 83.

MARSHALL, K. E., VADUKUL, D. M., DAHAL, L., THEISEN, A., FOWLER, M. W., AL-HILALY, Y., FORD, L., KEMENES, G., DAY, I. J., STARAS, K. & SERPELL, L. C. 2016. A critical role for the self-assembly of Amyloid-beta1-42 in neurodegeneration. *Sci Rep,* 6**,** 30182.

LAMBERT, M. P., VELASCO, P. T., CHANG, L., VIOLA, K. L., FERNANDEZ, S., LACOR, P. N., KHUON, D., GONG, Y., BIGIO, E. H., SHAW, P., DE FELICE, F. G., KRAFFT, G. A. & KLEIN, W. L. 2007. Monoclonal antibodies that target pathological assemblies of Abeta. *J Neurochem,* 100**,** 23-35.
